# Macro level influences on strategic responses to the COVID-19 pandemic – an international survey and tool for national assessments

**DOI:** 10.7189/jogh.11.05011

**Published:** 2021-07-01

**Authors:** Raheelah Ahmad, Rifat A Atun, Gabriel Birgand, Enrique Castro-Sánchez, Esmita Charani, Ewan B Ferlie, Izhar Hussain, Andrew Kambugu, Jaime Labarca, Gabriel Levy Hara, Martin McKee, Marc Mendelson, Sanjeev Singh, Jay Varma, Nina J Zhu, Walter Zingg, Alison H Holmes, the COMPASS (COntrol and Management of PAndemicS through Strategic analysis) study group

**Affiliations:** 1 National Institute for Health Research Health Protection Research Unit in Healthcare Associated Infection and Antimicrobial Resistance at Imperial College, Hammersmith Campus, London, UK; 2 Division of Health Services Research and Management, School of Health Sciences, City, University of London, Northampton Square, London, UK; 3 Department of Global Health and Population, Harvard T. H. Chan School of Public Health, Harvard University, Boston, Massachusetts, USA; 4 Centre d’Appui à la Prévention des Infections Associées aux Soins (CPias), Pays de la Loire, Nantes University Hospital, Nantes, France; 5 Division of Nursing, School of Health Sciences, City, University of London, Northampton Square, London, UK; 6 King’s Business School, King’s College London, Bush House, London, UK; 7 Institute of Business & Health Management, Dow University of Health Sciences, Karachi, Pakistan; 8 Infectious Disease Institute, Makerere University, Kampala, Uganda; 9 Department of Infectious Diseases, School of Medicine, Pontificia Universidad Católica de Chile, Santiago, Chile; 10 Unit of Infectious Diseases, Hospital Carlos G Durand, Buenos Aires, Argentina; 11 Department of Public Health, London School of Hygiene & Tropical Medicine, London, UK; 12 Division of Infectious Diseases & HIV Medicine, Department of Medicine, Groote Schuur Hospital, University of Cape Town, Cape Town, South Africa; 13 Department of Medicine, Amrita Institute of Medical Sciences, Amrita University, Kerala, India; 14 Africa Centres for Disease Control and Prevention, Addis Ababa, Ethiopia; 15 Infection Control Programme and WHO Collaborating Centre on Patient Safety, University of Geneva Hospitals, Geneva, Switzerland; 16 Division of Infectious Diseases and Hospital Epidemiology, University Hospital of Zurich, Zurich, Switzerland

## Abstract

**Background:**

Variation in the approaches taken to contain the SARS-CoV-2 (COVID-19) pandemic at country level has been shaped by economic and political considerations, technical capacity, and assumptions about public behaviours. To address the limited application of learning from previous pandemics, this study aimed to analyse perceived facilitators and inhibitors during the pandemic and to inform the development of an assessment tool for pandemic response planning.

**Methods:**

A cross-sectional electronic survey of health and non-health care professionals (5 May - 5 June 2020) in six languages, with respondents recruited via email, social media and website posting. Participants were asked to score inhibitors (-10 to 0) or facilitators (0 to +10) impacting country response to COVID-19 from the following domains – Political, Economic, Sociological, Technological, Ecological, Legislative, and wider Industry (the PESTELI framework). Participants were then asked to explain their responses using free text. Descriptive and thematic analysis was followed by triangulation with the literature and expert validation to develop the assessment tool, which was then compared with four existing pandemic planning frameworks.

**Results:**

928 respondents from 66 countries (57% health care professionals) participated. Political and economic influences were consistently perceived as powerful negative forces and technology as a facilitator across high- and low-income countries. The 103-item tool developed for guiding rapid situational assessment for pandemic planning is comprehensive when compared to existing tools and highlights the interconnectedness of the 7 domains.

**Conclusions:**

The tool developed and proposed addresses the problems associated with decision making in disciplinary silos and offers a means to refine future use of epidemic modelling.

As the COVID-19) pandemic continues to spread worldwide, countries affected earliest (China, Italy, France) are reviewing strategies for resurgences [1] and numerous inquiries are under way. The threat of the pandemic continues to challenge scientific and policy communities because of the inadequately understood characteristics of the virus and rapidity of spread. The absence of licensed vaccines or effective antiviral therapies mean that non-pharmaceutical interventions remain central to the response. Managing COVID-19 continues to raise societal dilemmas - breaking the chain of transmission through find, track, trace, isolate and support (FTTIS) systems amid concerns about privacy [2], widespread restrictions that themselves pose threats to the health of individuals and the economy [3,4]; implementing optimal clinical management protocols for those affected in health systems struggling to cope; and procuring supplies of the ultimate protection, a vaccine where there is uncertainty about its sustained efficacy and uptake even once it is available [5,6]. While we know that the pandemic is having an immediate impact on households and the wider economy, the long-term impact on health (Abbott, 2020; National Institute for Health Research, 2020), and social and economic consequences is yet to be quantified [7,8]. The nature and timing of response strategies has varied from strict and complete lockdown of cities and regions (eg, China) through a phased approach with implementation of track and trace taking primacy (Singapore, New Zealand) to a rejection of widespread restrictions (Sweden) or passive approaches (Brazil). In the current pandemic, the rationale for implementation of lockdown is not always explicit but ranges from protection of limited health care capacity (in Argentina and some central European countries), to stop-start implementation to mitigate catastrophic economic effects on the poor (Pakistan), and perception that the public may become complacent or fatigued (England). Other countries were able to mobilise technology-led approaches, such as Iceland with a biobank covering 6% of its population [9] or Germany, with extensive laboratory capacity [10]. Grouping countries by economic capability on usual measures (eg, gross domestic product (GDP)) does not seem to explain either the response strategy or the outcomes as measured by mortality [11]. Positive outliers based on economic determinants include Thailand and Greece, negative outliers include the UK and USA. Small, wealthy nations (eg, Qatar, Bahrain) have also had poorer outcomes than predicted by GDP. Other explanatory determinants such as prevailing societal norms found in democratic, or command and control and more autocratic political contexts also show a mixed picture with failures and successes associated with each [12,13], although countries with leaders pursuing populist policies seem to perform sub-optimally [14].

The current pandemic is on a scale not seen for a century, as to date other viruses with potential to cause pandemics have been contained regionally and, in some cases, suppressed in human populations (SARS-CoV-1, Zika). The learning from the published evidence base, can be grouped into three main categories. The first, a multitude of commentaries and opinion pieces; second, pandemic preparedness plans from national and international organisations, comprising overall guidance and detailed operational plans at local/regional level and a suite of predictive tools; and third, secondary or primary research to assess the contextual factors which influenced how the epidemic was managed. In a literature review of this third category [15] covering epidemics since 2000, only one of 19 studies examined all domains of an established strategic management framework spanning seven domains: political, economic, sociological, technological, ecological, legislative and wider industry (PESTELI) [16]; while 6 considered the sociological domain alone. Data used for analyses are largely confined to secondary sources with only 6 studies employing primary and secondary or mixed methods approaches, so, they do not benefit from multi-disciplinary inquiry and the necessary data triangulation. Moreover, the findings from analyses are often too late or inadequately disseminated and therefore do not effectively support learning and preparedness for future pandemics.

This study aimed to analyse perceived drivers and barriers during the 2020 pandemic, to start to address the evident gaps in coordinated reflection and communication [17] and respond to the call for real-time analysis during a pandemic [18]. We mobilise the PESTELI framework, routinely used in strategic management to assess the environment, but under-used in public and global health [15,19], and provide a comprehensive approach for guiding assessments of preparedness at the country level for the immediate and longer term.

## METHODS

### Study design and participants

We developed an online survey, which allowed anonymous submissions between 5 May 2020 and 5 June 2020 using Qualtrics (Provo, UT, USA). The Uniform Resource Locator (URL) link to the survey was widely distributed through a range of social media platforms reaching target groups, including the European Committee on Infection Control (EUCIC), International Society for Infectious Diseases, Réseau de Prévention des Infections Associées aux Soins (RéPias), and the international participants in a massive open online course on applying Social Science for tackling Antimicrobial Resistance. The survey could also be accessed through the project webpage (https://www.imperial.ac.uk/news/197358/strategic-planning-pandemic-management/) and was cascaded by email to collaborators. Participants consented to the use of their data in the study before accessing the questionnaire. The 31-item survey based on the seven domain PESTELI framework (electronic survey and Table S1 in the **Online Supplementary Document**) was pre-tested and piloted, translated and piloted again (the link to the electronic survey is provided in the **Online Supplementary Document**). The survey was made available in 6 languages (English, French, Spanish, Mandarin Chinese, Portuguese, and Korean). Participants were asked to score inhibitors (-10 to 0) or facilitators (0 to +10) impacting their country’s response to the COVID-19 pandemic in the seven domains included in the PESTELI framework and provide free-text comments. No word limit was imposed on responses. Upon completion of the survey, the participants could opt-in to be followed-up in six months (December 2020) and one year (May 2021).

### Data analysis and assessment tool development

**Figure 1:**
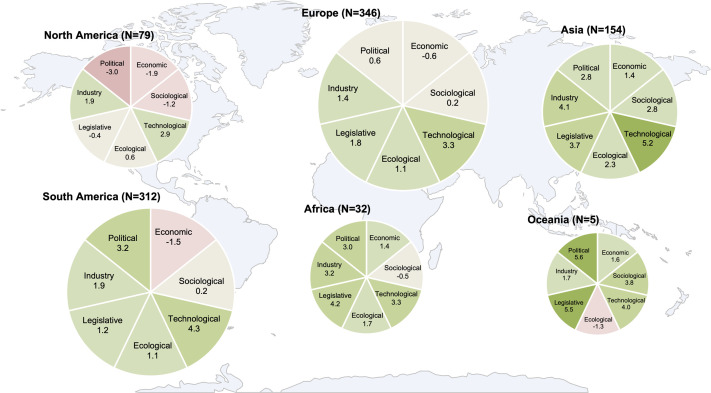
PESTELI domain scoring: by continent.

We report the perceived impact of each PESTELI domain as the percentage of participants scoring within the integer interval from -10 through to +10. The analysis was performed for the global survey data set (all countries) and then for individual countries with a sample size greater than 15: Argentina, China, Colombia, France, Mexico, Pakistan, Paraguay, South Africa, Spain, Thailand, the United Kingdom (UK), and the United States of America (USA). During the study period (5 May 2020 to 5 Jun 2020), China, France, Spain, and the UK had passed the peak of the pandemic, while Argentina, Colombia, Mexico, Pakistan, Paraguay, South Africa and the USA were still experiencing an increasing number of new daily cases (Figure S2 in the **Online Supplementary Document**). In addition, we calculated the mean score for each PESTELI domain for each continent. We performed data analysis using STATA 15 (StataCorp, College Station, TX, USA), and presented the mean scores in **Figure 1**, using six heat maps. The size of each heat map indicates the sample size of each continent. We used a colour gradient, deeper red, to illustrate a a high negative mean score, and deeper green to illustrate a higher positive mean score. We avoided erroneous attempts to establish statistical correlations as the outcomes of the pandemic are still unfolding in different parts of the world and this would be premature. Additionally, sampling was not representative of any particular professional group within countries.

We analysed all free text responses, blinded by country to reduce bias, sequentially, by language of response. Thematic analysis of all English responses was conducted by four researchers (RA, ECS, NJZ, GJCB), followed by analysis of the responses in other languages through automated translation to English in parallel with analysis within the source language. The themes were then validated by each multi-lingual co-investigator in the source language and differences resolved by discussion. While the themes were derived from barriers or facilitators or ‘both’, in the synthesised results these emergent factors are presented in a neutral language. This is to help formulate a construct or theme which can be objectively assessed. The qualitative themes are not presented by the country of respondent, as the themes are not necessarily representative of a country but designed to be inclusive of as many country contexts as possible. The respondent type is included in the illustrative quotes to provide some context.

The themes were validated with three types of triangulation (Thiessen & Denzin, 1970): 1) data triangulation - triangulated with findings from our literature review ([15]); 2) methodological triangulation - mapped against the existing pandemic preparedness tools, including the Global Health Security Index [20]; the International Health Regulations [21]; the Pandemic Influenza Preparedness (PIP) framework [22]; the Epidemic Preparedness Index [23]; 3) investigator triangulation - the themes were further validated by the authors comprising a range of multi-professional clinicians and experts (infectious diseases in high-, middle- and low-income settings, clinical pathology, health policy, health economics, organisational theory, sociology, digital health, and innovation adoption).

This thematic analysis resulted in the 103-item tool for assessing the macro-environment at the national level.

### Ethics

The study was approved by the Joint Research Compliance Office, Imperial College London (ICREC reference: 20IC5947).

## RESULTS

### Respondent demographic

The survey was completed by 928 respondents from 66 countries with a sample of 15 or more from 12 countries (Figure S2 in the **Online Supplementary Document**). This corresponded to a 68% response rate, calculated as the proportion of respondents going on to complete the survey of those that had opened the survey link. 588 respondents completed the survey in a language other than English. 67% of respondents work in health care organisations, of which, 85% are health care professionals. 15% work in academic institutes, 4% work in the private health care industry, 3% work in national or local government, 2% work in the private non-health care industry, 1% work in the international or national charity sector, 1% work in social care organisations and 7% work in ‘other’ organisations. 36% provided free text explanatory comments in addition to scoring. 41% provided an email address to be followed up.

### Quantitative scoring

**Figure 2:**
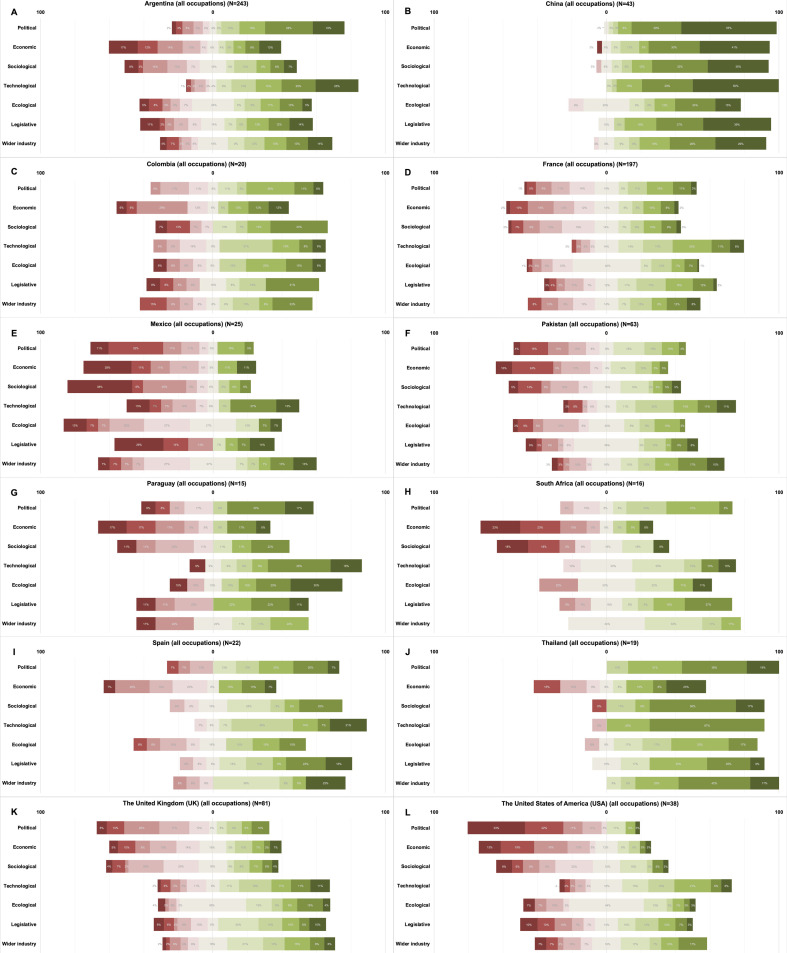
PESTELI domain scoring: by country. **Panel A.** Argentina. **Panel B.** China. **Panel C.** Colombia. **Panel D.** France. **Panel E.** Mexico. **Panel F.** Pakistan. **Panel G.** Paraguay. **Panel H.** South Africa. **Panel I.** Spain. **Panel J.** Thailand. **Panel K.** United Kingdom (UK). **Panel L.** United States of America (USA).

The aggregate mean scores by continent (**Figure 1**) including responses from all respondents provides a crude snapshot of perceptions. While each country may be vastly different, in terms of context and baseline infrastructure of health systems and economy, it is important to use this level of analysis when looking at pandemics as there are common issues in a threat of this nature. Notable inhibitors are ecological factors in Oceania, economic factors in South and North America, and additionally, political and sociological factors in North America. All continents register facilitating influences in the technological domain (range 2.9 to 5.2). Political factors are perceived positively in Oceania (5.6) and Africa (4.6) but neutral in Europe (0.6). The country breakdown for those with a sample size greater than 20 is shown in **Figure 2**.

The perceived facilitators and inhibitors for these nine countries are shown in Figure 2 and demonstrate the proportionate scoring within each domain. With the exception of China, overall, the lowest (-10) and highest (+10) are in the political and technological domains respectively.

### Thematic analysis

**Figure 3:**
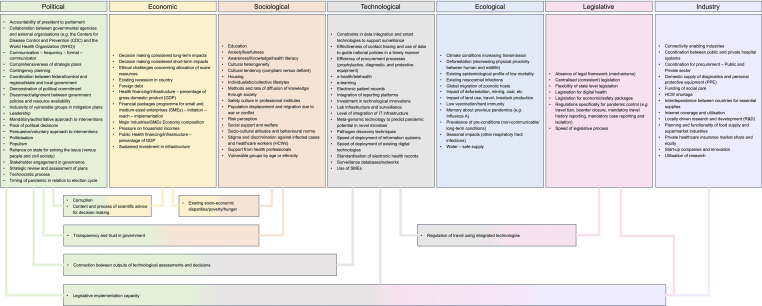
Qualitative themes in PESTELI domains.

The thematic analysis and synthesis resulted in 103 themes in total, with 7 crossing two or more domains (**Figure 3**). Though respondents were not directly asked to consider impacts of COVID- 19 on the macro-environment, this emerged from the data analysis and is reported.

The themes under the political and technological domains are now discussed in more detail, followed by the themes which cross two or more domains. Finally, the perceived impact of the COVID-19 pandemic back on the macro-environment are presented. In each of the countries, inhibiting political factors (with the exception of China) and facilitative technological factors attained the most extreme scores.

#### Political

The themes within the political domain were highly interconnected, with some factors acting as a precursor or determinant of other barriers within the political domain and other domains.

Lack of strategy and of detailed plans were seen as allowing politics to take primacy in decision making and politicisation of the pandemic.

“Because of the lack of effective pre-planning regarding healthcare policies and systems, political factors and leadership became a huge barrier” *(R 625– other)*

“The pandemic is used to make local politics, regardless of the risk to patients or doctors” *(R:HCP).*

Notably missing were comments about any political party but there was criticism of individual leaders and leadership.

“Our leadership at a national level has made the handling of this pandemic much more difficult. Getting mixed messages from the top causes people's behaviour to change based on incorrect information” *(R 819: Private industry)*

While mention of *leadership* is unsurprising, respondents were nuanced in their assessments of it, acknowledging challenges faced by leaders. However, the words *transparency* and *trust* featured prominently, whether lamenting their absence or celebrating their presence. Concerns were also raised about the mechanism, frequency, and format of the communication and the extent of scientific involvement and use of their recommendations by policy makers.

“It's a difficult time for anyone to be a leader, but to date, there has been a lack of transparency… the picture painted is always of a government having made all the right decisions at the right time. This is not the case. I am also concerned about the vilification of scientists who we now see being used as scapegoats for the decisions made” *(R 281: Private Healthcare*)

“…the responsibility of the government to take care of its citizens was also poor, which further affected the “trust” with its people” *(R133:HCP)*

“I believe that the public generally trust their government and follow the recommendations. This facilitates their adherence to the measures taken against the pandemic” *(R: HCP/academia/local government).*

Political structures and processes were identified as important factors, but in ways that varied. An example is the degree of centralisation of governance structures, with some devolved systems portrayed as fragmented and deviating from central government messaging or, on the other hand, allowing measures to be implemented rapidly that were appropriate to local context.

“Governors and Mayors are technically guided. Federal leadership is more political, confusing and many times not ethical and overall irresponsible” *(R: HCP).*

“Political system (set up) leads to huge advantage in managing the pandemic, enabled fast coordinated action and resource mobilisation” *(R: other)*

The pace of political decisions was an important factor in both centralised and devolved structures and rapid decision making was mostly seen as a facilitator, except when a government was perceived as corrupt (see corruption theme below).

The timing of the *pandemic in relation to the election cycle* was perceived as important. When an election was close, decisions were seen as driven by the need for popularity and short-term wins.

“The current government desperation to hold on to power has blind-sided the health of nation thus making their action reactive rather than proactive in addressing COVID-19” *(R9: HCP).*

“The pandemic came 4 months after a new government that received the country in very poor condition. But they made great decisions, putting everyone's health first” *(R: HCP).*

#### Technological

While the technological domain was scored as a facilitating factor in all countries, many of the themes address the potential, often unmet, of technology, and particularly its timely uptake and utilisation. Respondents referred to issues of existing technologies which needed to be scaled up and rolled out. Respondents also talked about simple technologies (eg, 3D printed visors) to more advanced technologies such as a networked surveillance/track and trace system.

“There should have been more in place prior to the pandemic rising to a level of emergency” *(R82: other).*

Weaknesses in the ability to implement and scale up technology were seen as a barrier, also influenced by issues in the economic domain.

“…but if resources had been allocated in advance, the measures would have been implemented quicker “without panic”. I think that before “innovating”, you have to be solid on the basics. In this case, this was not the case - race for masks and respirators, lack of staff in the teams, lack of space in establishments. Previous closures of beds, failure to respond to various requests from hospital staff as part of ‘economy’ measures in public hospitals” *(R: social care professional).*

However, the challenge was not limited to advanced technology. Access to basic equipment was also problematic.

“I am afraid that a belief in 'innovation' and technological solutions (e.g. tracing apps) as sexy silver bullets rather than a dedication to tried and tested public health tracking, tracing isolating work done by qualified people has severely damaged the ability to get on top of the disease. Likewise the fetishisation of favoured companies to produce novel ventilators rather than having good traditional existing capacity and supplier networks is problematic” *(R24: academic).*

“Technology helped in two ways, first in testing, treating, and tracing, and surveillance. The second for maintaining regular living activities, the Wechat health code (QR code red amber green system to tell if you were allowed to travel), food delivery, logistics, and online teaching, all helped” *(R: other).*

Additionally, while technology was noted as a facilitator for communication and e-health, this was counterbalanced by the potential for spread of misinformation.

“Difficulty by social networks, fake news and the fact that everyone gives their opinion” *(R: HCP).*

#### Content and process of scientific advice for decision making

A theme which transcends five domains is the content and process of the scientific advice and how and if this was used in decision making. The pace and mobilisation of research and development by academic and research institutes was seen to be a facilitative factor; but at the same time this was also seen to be hindered by political processes.

“Suppression of much of the scientific research from any field” *(R: other).*

In terms of content, there was some perceived difference in the weight placed on medical/scientific and economic issues when selecting ‘evidence’ to inform strategies to adopt. In terms of process, some reported that domestic and international scientific advice sometimes conflicted or were taken up selectively by policy makers.

“Political decisions are made without technical assistance or politicians manipulate technical aspects at their convenience” *(R: HCP)*

However, some policy makers were seen as searching for the best evidence.

“They care about the health of all of us. Trying not to collapse the health system. I like that he listens to the doctors” *(R: other)*

“Right decisions were made analyzing experiences from other countries” *(R: academic)*

#### Existing socio-economic disparities/poverty/hunger

Across high-, middle- and low-income countries, disparities and inequities affected almost every aspect of the response.

“COVID-19 will change the face of digital society. That said I speak from the perspective of a middle-income citizen who can afford the internet and a laptop. Only about +-50%-60% of our population use the internet…” *(R280: private health care industry)*

Socio-economic conditions that older people lived in were noted as barriers to effective responses, especially where informal support networks were weak and there were gaps in social services. Economic vulnerability impaired the public’s ability to adopt protective measures, either voluntarily or in compliance with national policies. This theme links also with *Inclusivity of vulnerable groups in mitigation plans*, under the political domain. Challenges facing health care workers struggling to sustain themselves and their families in the face of economic uncertainty was an inhibitor, just as it was for the general population and for patients in particular. Of the support available in the poorest countries:

“…this support comes nowhere close to closing the gaps in access to economic, education, healthcare” *(R25, academic)*

#### Legislative implementation capacity

Legislative inconsistency was in part attributed to fragmentation between central and local governments, while a failure to implement legislation was a barrier in several domains, sometimes exacerbated by a lack of structures to enforce legislation. The ability to enforce legislation was also shaped by social norms, *Cultural tendency (compliant vs defiant),* as well as the *Methods and rate of diffusion of knowledge through society,* and *perceptions of risk.* Employing persuasive approaches rather than, or in some combination with, mandatory measures (and the need for legislation) are considerations under the political domain.

#### Corruption

Corruption was cited as a barrier, including political and economic forms of corruption. Responses to the pandemic were impeded in the presence of endemic corruption.

“…is a common denominator, it is a drag…” *(R: national government)*

“…conditioned by elements of sectarianism and corruption” (R: Social care organisation)

“The National Legislative Power does not work. Laws that protect citizens are not passed, everything is done by the president through personal decrees. We are under an undemocratic, corrupt and authoritarian regime” *(R: HCP).*

The crisis was seen as creating opportunities for corruption and political opportunism.

“The government took advantage of the moment to monopolize everything in the executive branch and inactivate the Judiciary and Parliament…We have an undemocratic, authoritarian and corrupt government” *(R: HCP)*

#### Impact of COVID-19 on the domains

Impact of COVID-19 on the macro-environment was noted in three domains – sociological, ecological and industry.

*Sociological* – The negative effects include declines in mental well-being largely from physical and social isolation, especially among vulnerable groups. The rise in domestic violence and other crimes were cited. Positive impacts included opportunities for change in social norms and behaviours.

*Ecological* – Raised awareness about zoonoses and one-health, bringing an opportunity for change was noted. The dip in air pollution levels was also noted as a positive consequence. Negative impacts included risks to food security and agriculture and the increased use of disposable products.

*Industry* – Positive impacts on industry were initiation and adoption of new mechanisms and tools (eg, of molecular and other surveillance) and a stimulus to research and innovation. The negative impacts however are shortages of health care workers due to infection of frontline staff.

### Themes mapped against the existing pandemic preparedness tools

Mapping of existing tools against the PESTELI framework shows good coverage across the 7 domains. The high level assessment (Figure S3 **in the** **Online Supplementary Document**), however, does not reveal the missed opportunities apparent from a more granular assessment as demonstrated by the detailed mapping of the existing tools against the 103 themes developed through the qualitative analysis (Figures S4 and S5 in the **Online Supplementary Document**). While the political and ecological domains are well represented in existing tools, there are major gaps in coverage of themes in the remaining five domains.

## DISCUSSION

Existing predictive tools have so far been unable to adequately predict or explain the observed outcomes in terms of morbidity and mortality during this evolving pandemic. One of the most widely used tools, the Global Health Security Index, scored the USA and the UK top in the world [20]. Both countries however are dealing with challenges in bringing this pandemic under control, as are many other countries who scored poorly according to the index. The reductionist approach employed in that tool risks missing critical issues that have become apparent as the pandemic is unfolding. Analysis of this and other tools show the discrepancy between objective measures of preparedness and what actually happened [24,25]. It is clear that the political domain needs much more attention. We are urged that decisions be based on science and to consider consequences for vulnerable populations [26,27], but there is less consensus about what that evidence base should constitute. Additionally, the translation of evidence to policy is highly mediated by political factors. The effectiveness of overall system governance across health and social care systems is also key.

Several commentators have noted how those countries that experienced the 2015 outbreak of Middle East respiratory syndrome (MERS) - such as South Korea- and the 2003 severe acute respiratory syndrome (SARS) pandemic such as Laos, Cambodia, and Vietnam - have been able to introduce public health measures fairly quickly and sustain a strong response to the epidemic.

We suggest that a more holistic approach to pandemic preparedness is needed, focusing especially on the interconnectedness of the different domains discussed in this paper. An effective approach should also draw on how countries have dealt with other infectious diseases, such as tuberculosis (TB) and human immunodeficiency viruses/acquired immune deficiency syndrome (HIV/AIDS) to strengthen the response to this and emergent pandemics [7].

This is the first study to be conducted during a pandemic using a strategic management lens and can support contemporary assessment of how countries are responding and adapting. The timing of this study should allow assessment prior to probable resurgences as restrictions are lifted.

The themes within these domains are not all quantifiable – but there are already detailed instruments which can be mobilised to make the required assessments. Existing frameworks, include the TAPIC (Transparency, Accountability, Participation, Integrity, Capacity), to assess the quality of governance [28]; multiple vulnerability indexes have been developed to predict the impact on population sub-groups, including from the current COVID-19 pandemic in India and Kenya [29,30]; an e-health readiness assessment framework has been designed for low-resourced settings but is equally needed in high-income settings [31]; and recently, a survey assessing public confidence/trust in government [32]. Together, these respond to the identified need for metrics that can inform strategic planning [33].

Limitations of the study include potential differences in perceptions by those in countries at different stages of the pandemic, who have varied in their opportunity to draw lessons, and the variable sample size. In our analysis we weighted each category of PESTELI equally, regardless of the importance given by respondents. Also, we do not know how respondents considered the domains temporally, comparing new or old impacts from each of the domains. The free text qualitative data suggest both were considered. Of course, the scores are based on ‘perceptions’ of the respondents, which may reflect a reality (or not), and further exploration is needed to understand the foundations of these perceptions. We cannot know or control for respondent’s political ideology which may influence the results.

In terms of the tool, while there is no way of knowing which score would or should be optimal, detailed case studies can help formulate some benchmarks. Scores and their distributions may be compared within countries over time to track change, potentially compared to similarly organised/funded health care systems.

In conclusion, the future possibilities of using this tool are to assess pandemic preparedness ahead of possible resurgences of COVID-19 and during periods when the restrictions are eased or lifted. Immediate next steps for researchers and policy makers are to operationalise the tool in different country settings using the data which we already have and prospectively collect data which is needed. Additionally, and critically, such an assessment should be part of the cycle of review of national policies, integrated into systems strengthening and planning, not only for pandemic preparedness, but also in non-pandemic scenarios.

## Additional material

Online Supplementary Document
